# Memory Support System in Spanish: A Pilot Study

**DOI:** 10.3390/brainsci11111379

**Published:** 2021-10-21

**Authors:** Octavio A. Santos, Anapaula Rios-Rosales, Otto Pedraza, Caroline D. Bergeron, Melanie Chandler

**Affiliations:** 1 Department of Psychology, The Ottawa Hospital, Ottawa, ON K1Y 4E9, Canada; 2 Department of Psychiatry and Psychology, Mayo Clinic, Jacksonville, FL 32224, USA; anapaula.rios@icloud.com (A.R.-R.); pedraza.otto@mayo.edu (O.P.); chandler.melanie@mayo.edu (M.C.); 3 Division of Aging, Seniors and Dementia, Public Health Agency of Canada, Ottawa, ON K1A 0K9, Canada; caroline.bergeron2@canada.ca; 4 LIFE Research Institute, University of Ottawa, Ottawa, ON K1N 6N5, Canada

**Keywords:** subjective cognitive decline, cognitive rehabilitation, functional ability, behavioral intervention

## Abstract

Subjective cognitive decline (SCD) is prevalent in the general population, particularly among Hispanic adults. SCD increases the risk of mild cognitive impairment (MCI) and dementia. While non-pharmacologic interventions are recommended to mitigate cognitive decline and preserve daily function in SCD and MCI, such interventions are not readily available for Spanish-speaking Hispanic adults with SCD. This pilot study, preregistered at clinicialtrials.gov, aimed to develop a linguistically and culturally appropriate adaptation of an existing memory compensation intervention, the Memory Support System (MSS), from English to Spanish, and to gather data to assess its impact in this population. Twenty Spanish-speaking Hispanic adults with SCD and 16 support partners were recruited. Measures of treatment adherence, daily function, self-efficacy for memory, quality of life, mood, anxiety, and caregiver burden were assessed at baseline, treatment end, and 8-week follow-up. By treatment end, participants with SCD improved their general functional status, daily activities requiring organizational skills, and depression and anxiety symptoms. Partners reported improvement in anxiety by treatment end and in caregiver burden at follow-up. The MSS was successfully translated into Spanish and readily learned by participants with SCD and their partners. The MSS in Spanish may help with daily functioning and aspects of patient and family well-being.

## 1. Introduction

Over 20% of the general population reports subjective cognitive decline (SCD) [1], defined as cognitive concerns in the absence of objective evidence of cognitive impairment [2]. SCD dramatically increases with age [1], and individuals with SCD are at an increased risk of developing mild cognitive impairment (MCI) [3] and dementia [4]. In the absence of medications that improve cognition or delay the neurodegenerative process that often underlies SCD or MCI progression, it is now considered good practice to offer non-pharmacologic interventions [3], which may help to mitigate cognitive decline and preserve daily function in SCD [5] and MCI [6].

The Memory Support System (MSS) is an evidence-based, non-pharmacologic intervention for MCI. The MSS is a calendar and notetaking system to train persons with memory or cognitive decline to complete personal goals and relevant instrumental activities of daily living (IADLs) independently [7,8]. MSS training is associated with positive treatment adherence, sustained independence in IADLs, and improved memory self-efficacy for individuals with MCI compared to randomized controls [8]. Moreover, MSS training is associated with improved mood for the partners and caregivers of adults with MCI [8]. The MSS was developed and mainly used with predominantly White, English-speaking adults. Despite its clinical benefits, the MSS has yet to be implemented and evaluated among Spanish-speaking Hispanic adults.

The United States ranks second among the countries with the largest population of Spanish speakers [9], and the Spanish language represents the second most common spoken language [10]. In the USA, Hispanics are the largest ethnoracial minority group (17.8%), with 76% of them having Spanish as their primary language or who are Spanish–English balanced bilinguals [10]. Among Hispanics ages 65 or older, 21% are Spanish-only speakers [10]. Hispanics represent 8% of the growing older population of the USA [11]. Notably, 1 in 9 Hispanic adults experience SCD and, of this group, one-third report that SCD interferes with their IADLs and almost half report giving up daily activities or needing help with household tasks [12]. Consequently, there is a clinical need for greater non-pharmacologic interventions for this population. However, interventions for Hispanics should be provided in their preferred language and in a way that reinforces family values and overcomes cultural barriers [13]. This pilot study aimed to develop a linguistically and culturally appropriate adaptation of the MSS in Spanish and assess preliminary data on the impact of MSS training on program outcome measures in Spanish-speaking Hispanic adults with SCD and their support partners. Study methodology followed the initial pilot study using the MSS in English [7].

## 2. Materials and Methods

### 2.1. Clinical Trial Registration

The study was preregistered at ClinicalTrials.gov (number NCT03933085).

### 2.2. Participants

Inclusion criteria included (a) presence of SCD, defined as “self-perceived decline in any cognitive domain over time” according to Jessen and colleagues’ conceptual framework for research on SCD [14], or a diagnosis of MCI based on Peterson’s diagnostic criteria [15], as determined by a clinical neuropsychologist based on clinical interview and review of cognitive and functional screening measures (described next); (b) Clinical Dementia Rating global score of ≤0.5 [16]; (c) Linguistic History Form scores of ≥4 on items six to nine [16]; (d) Spanish Translation of the Dementia Rating Scale-Second edition score of ≥115 [17]; (e) available contact with a support partner ≥2 times weekly; and (f) absence or stable intake of nootropic(s) for ≥3 months. Exclusion criteria included (i) visual or hearing impairment; (ii) history of reading or writing disability sufficient to interfere with MSS training; or (iii) concurrent participation in another related clinical trial.

Potential participants included Spanish-speaking, community-dwelling adults residing in northern Florida, USA, identified between November 2018 and October 2019 via contact with Hispanic medical professionals, community associations including churches, and free local media (i.e., TV channel, newspaper, Facebook, hospital digital boards, flyers, monthly educational talks at Spanish churches and English as second language classes, and letters mailed and emailed to community and hospital physicians asking for referrals). At these meetings or events, potential participants were asked to sign up or register if they would like to be contacted for further information about the study. All eligible participants understood that they needed to be willing to participate in the maximum number of visits (i.e., 10 therapy sessions over 2–6 weeks).

**Figure 1:**
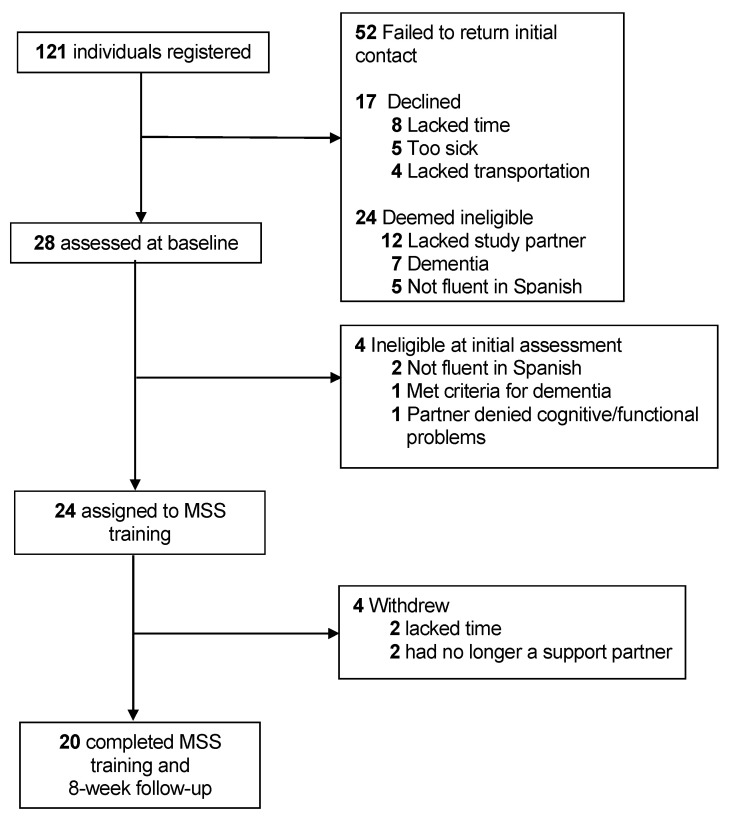
Study recruitment and retention.

A total of 121 individuals registered to potentially participate in the study. As shown in Figure 1, the final enrollment rate was 19.8% (*n* = 24), with an 83.3% (*n* = 20) retention rate for both the intervention and 8-week follow-up.

### 2.3. Procedure

#### 2.3.1. Translation/Cultural Adaptation

The institution’s international services provided a professional direct and back translation of MSS materials and measures. Initial translations were reviewed and evaluated by a bilingual and bicultural professional (O.A.S.) familiar with the intervention and materials and a bilingual and bicultural volunteer unfamiliar with the intervention and materials. Any translation discrepancies were resolved by the professional and volunteer in consultation with the institution’s international services. All materials were reviewed and administered to four Spanish-speaking community volunteers to field test and create the final version.

#### 2.3.2. MSS Training Paradigm

The MSS and its manualized training curriculum are described in detail in prior reports [7,8]. Briefly, the MSS is a two-page-per-day, pocket-sized calendar/note-taking system with three sections: (a) events; (b) to-do’s; and (c) journaling for logging important things to be remembered (e.g., news about family/friends). The MSS curriculum utilizes three training stages from learning theory [18]: acquisition (MSS is learned); application (participant is taught to apply MSS to his/her daily life); and adaptation (participant practices incorporating MSS into daily life). There are Intervention Plan/Questions (IPQs) covering topics to be learned in each training stage (see Figure S1 for an example of the two-page-per-day calendar in Spanish and Table S1 for the IPQs). Participants with SCD progress to the next MSS training stage after demonstrating 100% accuracy on the IPQs in a stage for two consecutive days. Each training session also provides orientation, modelling, practice use, and homework assignments to be completed with assistance from the support partner, as instructed. A licensed neuropsychologist (O.A.S.) served as the MSS trainer. MSS training consists of ten sessions of one hour for a dyad or two hours for a triad delivered over two or six weeks, starting 7–10 days after baseline assessment. The role of the support partner is to help the participant with SCD use the MSS and complete homework assignments. After the intervention, each participant and their partner completed a semi-structured interview, seeking suggestions for improving the MSS, teaching curriculum, and intervention logistics. MSS use was the only intervention used during training.

#### 2.3.3. Treatment Adherence

All participants with SCD were provided with the MSS calendar at the enrollment visit and instructed to “begin using the calendar to help with your memory”, without further verbal or written instruction. MSS adherence was assessed one week later at the beginning of the treatment, treatment end, and 8-week follow-up. Adherence scores are based on a scale from 0 to 10, with 10 being highest and a cut-off score of ≥7 points suggesting adherence with the MSS (i.e., frequent use of the MSS’ three aforementioned sections).

#### 2.3.4. Assessment Schedule and Outcome Measures

All written measures were provided in Spanish. At enrollment, participants with SCD and their support partners completed measures of language proficiency, acculturation, single-word reading, and cognitive and functional impairment. Participants and partners also completed measures of treatment adherence, IADLs, self-efficacy for memory, quality of life, mood, anxiety, and caregiver burden at baseline, treatment end, and 8-week follow-up. Specifically, language proficiency was measured by using the Linguistic History Form, a self-rated measure of the level of proficiency at speaking, reading, writing and listening in Spanish and English using a 7-point Likert-type scale ranging from 1 (almost none) to 7 (like native speaker) [16]. Acculturation was assessed using the Abbreviated Multidimensional Acculturation Scale, a 42-item self-report scale with 4-point Likert-type response options ranging from 1 (strongly disagree) to 4 (strongly agree) for the cultural identity subscales and from 1 (not at all) to 4 (extremely well/like a native) for the language and cultural competence subscales, three factors associated with acculturation in the USA and country of origin [19]. This scale has shown appropriate validity in Hispanic community members [19]. Single-word reading was measured by the Word Accentuation Test-Revised, which requires the individual to read aloud 50 low-frequency words with irregular accentuation and printed in uppercase letters without their graphic accents [20]. This measure is highly and significantly correlated with traditional measures of estimated premorbid function (e.g., Vocabulary from the Wechsler Adult Intelligence Scale) and has shown suitable psychometric properties [20].

Cognitive and functional impairment was assessed by the Spanish version of the Clinical Dementia Rating Scale, a 5-point scale that assesses three domains of cognition (memory, orientation, and judgment/problem solving) and three domains of function (community affairs, home/hobbies, and personal care) through a semi-structured interview of the patient and a reliable informant, with scores of ≤0.5 suggesting normal or questionable impairment [16]. Cognition was assessed by the Mini-Mental State Examination [16] and the Spanish Translation of the Dementia Rating Scale-Second edition [17]; the latter consists of five subscales (attention, initiation/perseveration, construction, conceptualization, and memory) and a total score normed based on age and education in Spanish-speaking healthy controls in the USA [17]. The Mini-Mental State Examination and Spanish Translation of the Dementia Rating Scale are reliable and valid instruments for the assessment of cognitive impairment in Spanish older adults [16,17].

Mood was measured by the Spanish version of the Center for Epidemiologic Studies Depression Scale, a 20-item self-report scale with 3-point Likert-type response options ranging from 0 (rarely or none of the time or less than one day) to 3 (most or all of the time or 5–7 days) [21]. Anxiety was measured by the State-Trait Anxiety Inventory by the Resources for Enhancing Alzheimer’s Caregiver Health project, a 10-item rating scale modified from the State-Trait Anxiety Inventory [22]. Quality of life was measured by the Quality of Life in Alzheimer Disease instrument, a 13-item measure developed for individuals with dementia that has been utilized in MCI and with care partners who rate their relationships, concerns about finances, physical condition, mood, energy level, memory, aspects of daily functioning, and overall life quality on a 4-point scale ranging from 1 (poor) to 4 (excellent) [23]. Self-efficacy for memory was assessed using modified, selected items from the Chronic Disease Self-Efficacy Scales [24]. The 9-item Self-Efficacy in Mild Cognitive Impairment Scale includes specific items relevant to MCI (i.e., “your memory/cognitive difficulty” rather than more general references to “your health condition”) [24].

IADLs were assessed by the informant-based Functional Assessment Questionnaire [16] and the memory and executive functioning subscales of the informant-based Everyday Cognition questionnaire [25]. The Spanish version of the Functional Assessment Questionnaire measures the ability of older adults, over the previous four weeks, to perform ten IADLs rated on a 4-point scale ranging from 0 (“normal”) to 3 (“dependent”) [16]. The Spanish version of the Everyday Cognition questionnaire assesses a participant’s ability to perform everyday tasks in different areas (memory, language, visuospatial abilities, and executive functioning), with suitable accuracy and correlation with other tests measuring daily and cognitive functions [25]. Only the Everyday Cognition’s subscales of memory and executive functioning (planning, organization, and divided attention) were used. Performance-based IADLs were assessed using the Pillbox Test, an ecologically valid measure to assess executive functions through the real-time assessment of medication management [26]. The Pillbox Test has shown suitable criterion-related validity and convergent validity with other measures of executive functions [26]. Caregiver burden was assessed by the Caregiver Burden Inventory Short-Form, a 12-question inventory that measures the degree of stress experienced by family caregivers concerning the effect of the participant’s disability on care partners’ lives [27]. The Clinical Dementia Rating, Linguistic History Form, Functional Assessment Questionnaire and Mini-Mental State Examination are from the Spanish language Uniform Data Set from the National Alzheimer’s Coordinating Center [16].

### 2.4. Analysis

A within-subject, multiple baseline design was used. Data quality was investigated by calculating item and total score distribution, and missing data per item with descriptive statistics. Changes in treatment adherence, IADLs, mood, anxiety, quality of life, self-efficacy, and caregiver burden were assessed using pairwise *t*-test comparisons. Change in percentage of MSS adherence and Pillbox Test pass scores were analyzed using the McNemar test. A binary logistic regression was performed to ascertain the effects of age, education, acculturation, and outcome measures (IADLs, self-efficacy, quality of life, mood, anxiety, and caregiver burden) on the likelihood that participants with SCD were treatment adherent (dependent variable). Effect sizes were calculated by the mean and standard deviation of the change scores: ES = Mdiff/SDdiff. Because this study represents a preliminary investigation, correction of the familywise error rate was considered but deemed too stringent. Statistical analyses were conducted using SPSS Statistics 23.0 (IBM Corp, Armonk, NY, USA). *p* values of ≤0.05 were regarded as statistically significant results for all analyses.

## 3. Results

All 20 participants reported SCD, which was corroborated by a support partner. No participant had a formal MCI diagnosis or was deemed to meet definite MCI criteria based on a brief clinical interview and screening test results. Thirteen participants (65%) learned about the study from church, five participants (25%) from digital boards, and two participants (10%) from Facebook. Only two participants with SCD (10%) were initially concerned about the number of MSS training sessions; these participants were also the only ones who were employed whereas the other 18 participants with SCD (90%) were retired.

Among the 20 individuals with SCD, 13 had their adult child/family member as their support partner, six had a spouse as their support partner, and one had a paid caregiver as their support partner. There were 16 support partners in total and, among those, four served as a partner for more than one participant. Therefore, there were 12 dyads (one participant and one partner) and four triads (two participants and one partner). Table 1 displays the characteristics of the final sample of 20 participants with SCD and 16 respective support partners. No significant differences were noted in demographic characteristics or baseline performance between those who completed and did not complete training (see Table S2 for these non-significant results).

As shown in Table 2, participants with SCD were significantly more likely to adhere to the MSS in Spanish after training than before training, *t*(19) = −16.778, *p* < 0.001, *d* = −3.75. MSS adherence remained significantly high at 8-week follow-up compared to baseline, *t*(19) = −11.423, *p* < 0.001, *d* = −2.5. The percentage of participants with SCD adherent to the MSS in Spanish at each assessment point was as follows: 0% at baseline, 70% at treatment end, and 65% at follow-up (McNemar test *p* < 0.001).

Regarding IADLs (see Table 2), the Functional Assessment Questionnaire scores were significantly lower at the end of training (*p* = 0.004) and 8-week follow-up (*p* = 0.015) compared to baseline. The Everyday Cognition total scores were significantly lower at follow-up compared to baseline (*p* = 0.008). In particular, the Everyday Cognition memory subscale scores were significantly lower at follow-up compared to baseline (*p* = 0.022). The Everyday Cognition organization subscale scores were significantly lower at the end of training (*p* = 0.008) and follow-up (*p* = 0.013) compared to baseline. The percentage of participants with Pillbox Test passing scores after training was not significant. No significant differences were found from baseline to treatment end or follow-up on the Everyday Cognition planning or divided attention subscales.

No significant differences were found from baseline to treatment end or follow-up in the participants with SCD’s self-efficacy or quality of life scores. Regarding mood (see Table 2), the participants with SCD’s reported depressive symptoms were significantly lower at treatment end compared to baseline (*p* < 0.001). No significant differences were found from baseline to treatment end or follow-up in the partners’ reported depressive symptoms. Compared to baseline, anxiety symptoms were significantly lower only at treatment end for both participants with SCD (*p* = 0.001) and partners (*p* = 0.02). Caregiver burden was significantly lower only at follow-up compared to baseline (*p* = 0.009). Binary logistic regressions indicated no association between MSS adherence and age, education, acculturation, and functional and psychological outcomes.

Results from the semi-structured interviews after MSS training completion revealed that the MSS calendar had a positive impact on the lives of participants with SCD and their support partners. Specifically, participants with SCD reported better tracking of appointments, medications and daily tasks, increased independence, and a return to social activities at treatment end and follow-up. While not reflected on the formal self-efficacy questionnaire, all participants with SCD qualitatively reported increased self-confidence in doing daily activities and quality of life, and decreased anxiety at treatment end and follow-up. Additionally, 17 participants with SCD (85%) reported decreased sadness at treatment end and follow-up; the three other participants with SCD (15%) reported ongoing sadness in the context of recent life changes, including an inability to pursue previously practiced sports due to mobility problems, loss of friends or family members, and change in job conditions. Among support partners, 15 (93.8%) reported increased quality of life and decreased caregiver burden and one (6.3%) reported increased caregiver burden in the context of adjusting to new job demands. Fourteen participants with SCD (70%) regularly used all three sections of the MSS calendar (events, to-do’s, and journaling), but a notable minority (30%) used only the events and to-do’s sections. While all but two participants with SCD (10%) thought that the IPQs were monotonous, they also expressed the importance of these practice questions to become familiar with the MSS. All participants reported that the MSS training fulfilled their expectations and would recommend the intervention to others.

## 4. Discussion

We created a Spanish translation and cultural adaptation of the MSS and piloted it in 20 primary Spanish speakers with SCD. To the best of our knowledge, this is the first version of this compensatory memory notebook system for memory-impaired adults translated and validated in Spanish. Participants with SCD were successfully able to adopt the MSS in Spanish. Adherence significantly improved with training but was not as high as the adherence level achieved in the original White MCI sample (8% baseline, 95% treatment end, 84% 8-week follow-up) [7], most likely because patients with MCI routinely used the journaling section of the MSS while the majority of participants with SCD used the events and to-do sections. It is possible that participants with SCD did not perceive a comparable benefit of journaling about their day as did participants with established episodic memory loss (amnestic MCI). Feedback from participants helped us fine-tune and improve the translation of the MSS (e.g., referring to a pharmacy bottle as “pomo” in Puerto Rico and “frasco” in Colombia). It is hoped that this cultural adaptation will help improve adoption and adherence in future groups.

Treatment end and 8-week follow-up outcomes in this feasibility group were quite promising. By treatment end, participants with SCD showed significant improvement in functional status overall, and particularly in daily activities requiring organizational skills. Improvement in overall functional ability and organizational skills were maintained at follow-up, and improvement in memory-related IADLs was significant at follow-up. Importantly, given our small sample, these changes reflect moderate and nontrivial effect sizes. In our experience in past trials in Whites, it is not uncommon for effects to not be realized until some time goes by after the end of training to allow families time to see the impact on day-to-day life. Thus, the delayed impact on memory-related IADLs was not surprising. The improvements seen in IADLs are consistent with results from prior trials of the MSS [7,8].

Additionally, participants with SCD showed a significant improvement in depression and anxiety symptoms by treatment end (large effect sizes), while support partners reported significant improvement in anxiety (moderate effect size). These improvements in psychological functioning were not maintained at the 8-week follow-up despite reporting improvement in mood and anxiety during the semi-structured interviews; however, caregiver burden became significantly better by follow-up. This delayed impact on caregivers was also observed in previous work in Whites [8].

Contrary to past results showing improvement in self-efficacy for memory in patients with amnestic MCI [8], participants with SCD did not show a significant change in self-efficacy despite reporting in the interview that they felt more confident and verbalized significant improvement in overall functional status. This may have been related to the fact that self-efficacy scores were relatively high at baseline (79.7 ± 14.2), with little range on the measure to reflect improvement. Moreover, participants SCD were not circumscribed to memory complaints in this sample but included perceived problems with concentration, word-finding, planning, organizing, and multitasking. Thus, the lack of significance may reflect limitations in the memory-based, self-efficacy questionnaire to capture the positive reports heard in the interviews.

This feasibility trial was not without limitations. We used a treatment-only, quasi-experimental design (e.g., not a randomized control trial) with a small sample size underpowered to examine efficacy. Still, moderate to large effect sizes were achieved on outcomes of daily and psychological function that can help serve as guide-posts for powering future efficacy trials. As this is a pilot study, the results should be taken with caution until they are replicated with larger samples. Selection of participants was based on a brief interview and cognitive and functional screening tests reviewed by a neuropsychologist to determine presence of SCD. The scope of our assessment battery in this study limited our ability to make a confident MCI diagnosis. Using a medical or neurological exam along with detailed neuropsychological assessment would have helped us differentiate potential MCI from SCD participants. It is important to highlight the fact that, despite our professional and community outreach efforts, we were unable to recruit Spanish-speaking individuals diagnosed with MCI. This likely reflects a larger societal issue of lack of access to health care services available for non-English speakers in the USA [28]. Most of our recruits came from local churches. Anecdotally, only two participants in this trial had even talked to their health providers about their cognitive concerns. This is consistent with research showing that Hispanics are more likely to report poor communication about their symptoms with their health providers in the USA [29]. Interestingly, almost half of study participants with SCD were either spouses or siblings who wanted to participate together with a common support partner; the latter also being a family member, such as an adult daughter/niece or son/nephew. Given that the family is often regarded as the most important institution for Latinos regardless of country of origin, social class, or length of residence in the USA [30], the MSS or other intervention programs that are more family-oriented and that promote “familismo” may be more effective or favored in this population over other individual-based interventions.

## 5. Conclusions

As evidence grows regarding the efficacy of non-pharmacologic interventions in treating the symptoms associated with memory loss in SCD and MCI, these treatments should be made available to minority populations in the USA, who may be disproportionately burdened with the risk of developing dementia. This project provides one such translation and adaptation, showing that primary Spanish speakers with SCD can learn a memory compensation curriculum and use it on their own. Using the MSS in Spanish may also improve general functional status and caregiver burden. Results from this pilot study are promising and require replication.

## Figures and Tables

**Table 1:** Participant characteristics and baseline test results.

Characteristic	Participant with SCD (*n* = 20) Mean (SD)	Support Partner (*n* = 16) Mean (SD)
Age, years	66.80 (10.29)	49.44 (16.76)
Women, no. (%)	16 (80)	11 (68.75)
Education, years	14.80 (3.59)	15.94 (1.73)
Marital status, no. (%)		
Married	11 (55)	13 (81.25)
Divorced/Separated	4 (20)	1 (6.25)
Widowed	4 (20)	0 (0)
Single/never married	1 (5)	2 (12.50)
Ethnicity, no. (%)		
Hispanic/Latino	20 (100)	15 (93.75)
White	0 (0)	1 (6.25)
Primary language, no. (%)		
Spanish	19 (95)	9 (56.25)
English	0 (0)	2 (12.50)
Both	1 (5)	5 (31,25)
Language proficiency ^a^		
Spanish	6.26 (0.99)	6.23 (1.13)
English	3.95 (1.78)	5.39 (1.84)
Acculturation ^b^		
American culture	2.60 (0.68)	3.03 (0.83)
Culture of origin	3.30 (0.46)	3.39 (0.44)
Test score results		
WAT ^c^	34.20 (9.58)	29.13 (11.22)
Participant’s memory functioning ^d^	4.8 (1.7) ^^^	4.19 (1.80) ^^^^
MMSE ^e^	28.6 (1.4)	29.56 (0.51)
ST-DRS-2 ^f^	133.2 (7.0)	
Place of birth, no. (%)		
Puerto Rico	9 (45)	2 (12.50)
Colombia	5 (25)	1 (6.25)
Cuba	3 (15)	2 (12.50)
Argentina	1 (5)	3 (18.75)
Peru	1 (5)	1 (6.25)
Nicaragua	1 (5)	1 (6.25)
USA Mainland	0 (0)	6 (37.50)
Gross household annual income, no. (%)		
<$25,000	6 (30)	0 (0)
$25,000–49,999	6 (30)	4 (25)
$50,000–74,999	3 (15)	3 (18.75)
$75,000–99,999	2 (10)	2 (12.50)
$100,000–149,000	1 (5)	4 (25)
>$150,000	1 (5)	1 (6.25)

Note. MMSE = Mini-Mental State Examination [16]; ST-DRS-2 = Spanish Translation of the Dementia Rating Scale-Second edition [17]; WAT = Word Accentuation Test [20]. a Range is 1 to 7; higher score indicates greater self-reported language proficiency. b Range is 1 to 4; higher score indicates greater self-reported acculturation. c Range is 1 to 50; higher score indicates greater number of correctly read infrequent, prosodically accented Spanish words. d Range is 0 to 10; higher score indicates worse self-reported memory functioning. ^ Rating by participant with SCD and ^^ of participant’s memory functioning by partner. e Range is 0 to 30; higher score indicates better global cognition. f Range is 0 to 144; higher score indicates better global cognition.

**Table 2:** Outcome variables for participants with SCD and their support partners.

	Baseline (T1) Mean (SD)	Treatment End (T2) Mean (SD)	Follow-up (T3) Mean (SD)	T1–T2 Cohen’s *d*	T2–T3 Cohen’s *d*	T1–T3 Cohen’s *d*
Participant with SCD						
Adherence ^a^	1.20 (0.62)	7.80 (1.47)	7.45 (2.31)	−3.75 ***	0.13	−2.55 ***
Daily functioning						
FAQ ^b^	2.65 (2.54)	0.80 (1.40)	1.10 (1.68)	0.72 **	−0.32	0.59 *
ECog Total ^c^	39.80 (13.64)	35.80 (11.49)	31.05 (7.89)	0.28	0.54 *	0.67 **
ECog memory ^d^	13.65 (3.86)	12.55 (4.39)	11.50 (3.56)	0.24	0.37	0.56 *
ECog planning ^e^	6.65 (2.08)	6.60 (2.62)	6.40 (2.04)	0.02	0.08	0.13
ECog organization ^f^	8.6 (3.22)	7.30 (2.75)	7.15 (2.03)	0.66 **	0.07	0.61 *
ECog divided attention ^g^	7.30 (3.26)	6.50 (2.40)	6.00 (2.03)	0.34	0.25	0.42
Pillbox Test, no. (%) ^h^	10 (50)	13 (65)	13 (65)			
Self-efficacy for memory ^i^	79.65 (14.17)	82.35 (9.70)	83.65 (6.50)	−0.29	−0.21	−0.40
Quality of life ^j^	37.70 (5.62)	39.30 (4.46)	38.55 (5.48)	−0.44	0.19	−0.18
Depression ^k^	11.95 (12.38)	7.85 (10.99)	10.95 (11.34)	0.99 ***	−0.52 *	0.11
Anxiety ^l^	20.10 (6.91)	16.3 (5.78)	18.5 (7.09)	0.84 **	−0.37	0.28
Support partner						
Caregiver burden ^m^	8.20 (6.83)	6.80 (7.11)	5.80 (6.68)	0.39	0.3	0.65 **
Quality of life ^j^	40.50 (6.58)	40.56 (6.22)	41.31 (5.85)	41.31 (5.85)	−0.18	−0.21
Depression ^k^	8.06 (7.51)	6.88 (7.24)	6.06 (6.75)	0.24	0.15	0.37
Anxiety ^l^	19.50 (6.83)	16.88 (6.35)	17.5 (5.49)	0.65 *	−0.11	0.32

Note. Comparison of outcome score in combined group at baseline (T1), treatment end (T2), and 8-week follow-up (T3) (paired *t*-test (*df* = 19: * *p* < 0.05; ** *p* < 0.01; *** *p* < 0.001)). ECog = Everyday Cognition modified version [25]; FAQ = Functional Assessment Questionnaire [16]. ^a^ Range is 0 to 10; higher score indicates greater treatment adherence. ^b^ Range is 0 to 30; higher score indicates worse informant-rated instrumental activities of daily living. ^c^ Range is 23 to 92; higher score indicates worse informant-rated ability to perform everyday tasks involving memory and executive functioning. ^d^ Range is 8 to 32; higher score indicates worse informant-rated ability to perform memory-related everyday tasks. ^e^ Range is 5 to 20; higher score indicates worse informant-rated ability to perform planning-related everyday tasks. ^f^ Range is 6 to 24; higher score indicates worse informant-rated ability to perform organization-related everyday tasks. ^g^ Range is 4 to 16; higher score indicates worse informant-rated ability to perform everyday tasks involving divided attention. ^h^ Number and percentage of those who passed the Pillbox Test. ^i^ Range is 9 to 90; higher score indicates better self-reported self-efficacy in managing daily activities. ^j^ Range is 13 to 52; higher score indicates better self-reported quality of life. ^k^ Range is 0 to 60; higher score indicates worse self-reported depressive symptoms. ^l^ Range is 10 to 40; higher score indicates worse self-reported anxiety symptoms. ^m^ Range is 0 to 48; higher score indicates worse self-reported caregiver burden.

## Data Availability

Data are available from the corresponding author upon reasonable request.

## Supplementary Materials

The following are available online at https://www.mdpi.com/article/10.3390/brainsci11111379/s1, Figure S1: Example of Two-Page-Per-Day Calendar in Spanish, Table S1: Intervention Plan/Questions (IPQs), Table S2: Demographics and Baseline Performance Non-Significant Results.
