# Supplementary material for: Memory Support System in Spanish: A Pilot Study
Source: Brain Sci. 2021 Oct 21;11(11):1379. doi: 10.3390/brainsci11111379 (PMC8615490; doi:10.3390/brainsci11111379)
Supplement: Supplementary file 1 [file brainsci-11-01379-s001.zip › brainsci-1347033-supplementary.pdf]

Figure S1: Example of Two-Page-Per-Day Calendar in Spanish

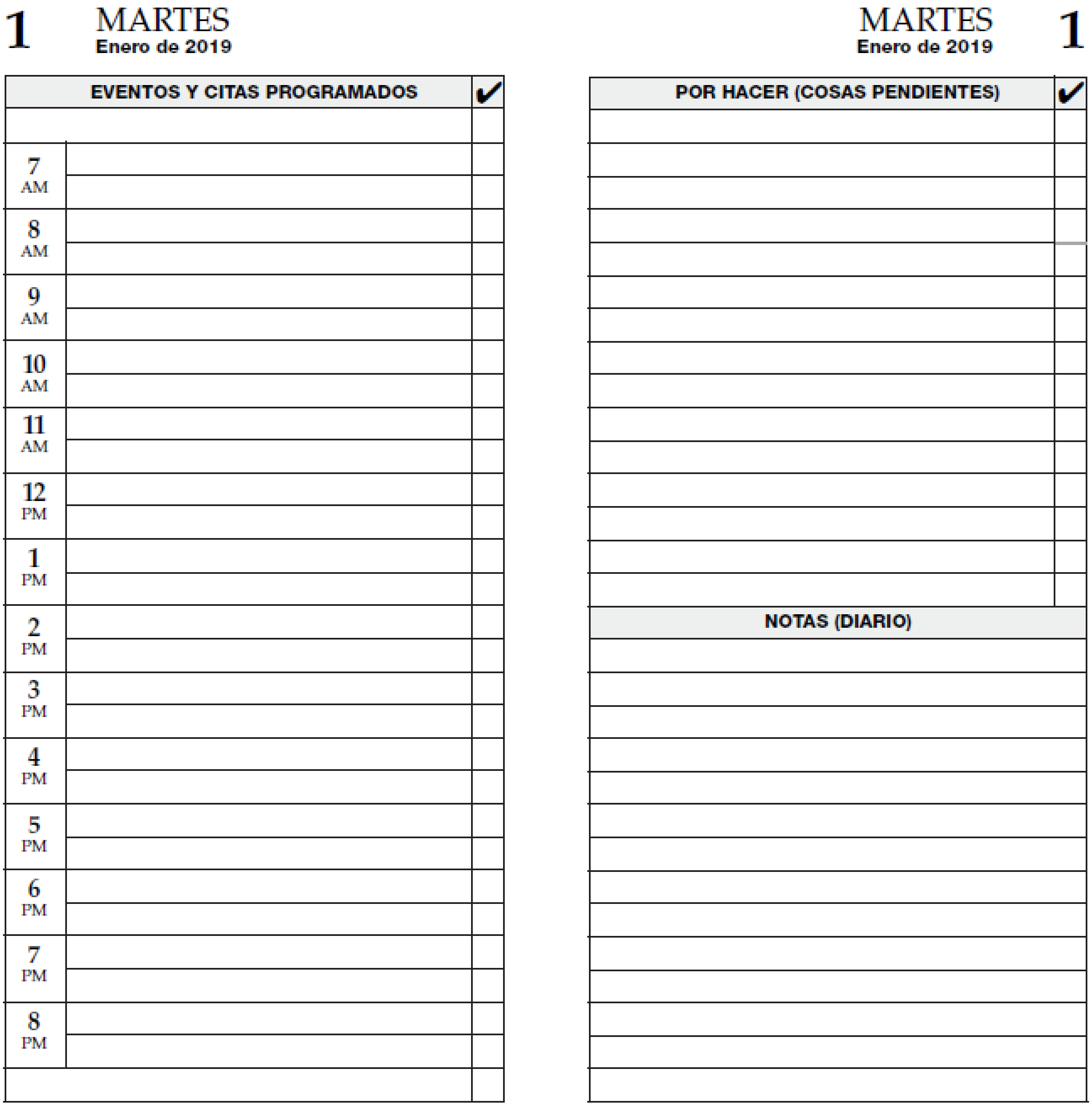

Table S1: Intervention Plan/Questions (IPQs)

Table S1: Intervention Plan/Questions (IPQs)

Preguntas de Aplicación

Nombre: ___________________________________

Puntuación: 0 – no puede mostrarla. **1** - necesita una señal directa. **2** – necesita señal(es) indirecta(s). **3** – da la respuesta o la señala sin ninguna señal del compañero. Empiece las preguntas con “mire en su agenda” …

**Fecha de hoy:**											
**Por favor abra la agenda en el día de hoy. ¿Dónde está la fecha?** *Mira hacia la parte superior de cada página.*											
**¿Cuáles son las 3 secciones principales de la agenda para cada día?** *Dice las 3 secciones.*											
**¿Dónde escribe las citas programadas para una determinada hora?** *Sección de “Eventos y citas programados” (junto a la hora).*											
**¿Dónde escribe una nota acerca de algo?** (o sea, la información que puede ser útil más adelante). *Sección de “Notas (diario).*											
**¿Dónde debe escribir una lista de las tareas que necesita hacer (**pero no en un determinado momento**)?** *Sección “Por hacer (cosas pendientes)”.*											
***¿Cómo debe marcar una tarea ya completada?**											
*** Cuando use esta agenda, usted debe poner una estrella junto a algo de alta prioridad. ¿Cómo debe marcar algo que es de alta prioridad?**											
*** Quiero que durante el período de instrucción, vea la agenda por lo menos 3 veces diarias. ¿Cuántas veces debe mirar la agenda diariamente como mínimo?**											
**Puntuación total** (24 puntos posibles)											

*Indica que esto debe hacerse con el formato de aprendizaje sin errores y recuperación espaciada. La puntuación es solamente de 3 o de 0, sin ninguna señal. No permita que el paciente adivine.

Preguntas de Aplicación

Nombre: ________________________________

Puntuación: 0 – no puede mostrarla. **1** - necesita una señal directa. **2** – necesita señal(es) indirecta(s). **3** – da la respuesta o la señala sin ninguna señal del compañero. Empiece las preguntas con "mire en su agenda" …

**Fecha de hoy:**									
**¿Cuál es la fecha de hoy?** *Se remite a la agenda para asegurarse.*									
**Vea el día de hoy: ¿qué citas o eventos tiene programados?** *Revisa los eventos programados y las citas.*									
**Vea el día de hoy: ¿qué tiene en la lista de cosas pendientes por hacer** (aquello que no está programado para un momento específico)**?** *Revisa la sección de "Por hacer (cosas pendientes)".*									
**Vea el día de hoy: ¿puede marcar una estrella junto a algo que sea de alta prioridad? (O ¿qué marcó como de alta prioridad con una estrella?** *Revisa e identifica las prioridades, marca en caso necesario.*									
**Vea el día de ayer: ¿llegó a todas sus citas e hizo todo lo que tenía pendiente?** *Revisa las cosas marcadas como hechas.*									
**Vea el día de ayer: ¿quedó algo para el día siguiente porque no lo hizo?** *Revisa la lista y anota en el siguiente día lo que no terminó.*									
**Vea el día de ayer: ¿escribió alguna nota?** *Se remite a la sección de "Notas (diario)".*									
***Usted decidió ver su agenda a (tal hora de la mañana), (tal hora de la tarde), (tal hora de la noche). ¿Cuándo revisa la agenda a diario?**									
**Puntuación total** (24 puntos posibles)									

*Primero, haga que el paciente escoja 3 momentos del día que tienen sentido para él (ella) y ocurren por la mañana, por la tarde y por la noche (p. ej.: comidas). De la respuesta con el formato de aprendizaje sin errores y recuperación espaciada. La puntuación es de 3 o de 0, sin ninguna señal. No permita que el paciente adivine.

Preguntas de Adaptación

Nombre: ___________________________________

Puntuación: 0 – no puede mostrarla. **1** - necesita una señal directa. **2** – necesita señal(es) indirecta(s). **3** – da la respuesta o la señala sin ninguna señal del compañero. Empiece las preguntas con "mire en su agenda" …

**Fecha de hoy:**									
**¿Con cuánta frecuencia mira usted su agenda todos los días?** *Demuestra uso continuo, al menos 3 veces diarias.*									
**Mire al último día (***escoja cualquier día de semana***), ¿llegó a todas sus citas y cómo sabe que lo hizo?** *Demuestra que usa el sistema de marcar las cosas como hechas.*									
**Mire al último (***escoja cualquier día de semana***), ¿qué completó de su lista de cosas pendientes por hacer?** *Por hacer (cosas pendientes), marca las cosas hechas.*									
**Mire al último día (***escoja cualquier día de semana***), ¿dejó para después alguna tarea que no terminó?** *Anota en el día siguiente lo que quedó incompleto.*									
**Mire en la última semana y deme el ejemplo de alguna nota que escribió** (información que deseaba recordar más tarde). *Sección de "Notas (diario)".*									
**¿Qué hizo el último fin de semana?** *Se remite a la agenda.*									
**Mire al** (*escoja un día que tenga algo que usted sabe es de alta prioridad, dentro de lo posible)*, **¿qué cosa es de alta prioridad?** *Busca la estrella.*									
**Mire al (***escoja un día después de hoy***), ¿está libre para ir a (***escoja:* **almorzar, cenar, al cine)?** *Se remite a "Eventos y citas programados".*									
**Puntuación total** (24 puntos posibles)									

Table S2. Demographics and Baseline Performance Non-Significant Results.

Characteristic	Participant with SCD who completed MSS training (*n*=20) Mean (SD)	Participant with SCD who did not complete MSS training (*n*=4) Mean (SD)	*t*-test or Chi-Square/Fisher exact test	*p* value
Age, years	66.80 (10.29)	64.5 (7.59)	0.42	0.68
Women, no. (%)	16 (80)	3 (75)	0.05	0.82
Education, years	14.80 (3.59)	12.5 (4.73)	1.11	0.28
Marital status, no. (%)			0.30	0.96
Married	11 (55)	2 (50)		
Divorced/Separated	4 (20)	1 (25)		
Widowed	4 (20)	1 (25)		
Single/never married	1 (5)	0 (0)		
Ethnicity, no. (%)				
Hispanic/Latino	20 (100)	4 (100)		
White	0 (0)	0 (0)		
Primary language, no. (%)			0.21	0.65
Spanish	19 (95)	4 (100)		
English	0 (0)	0 (0)		
Both	1 (5)	0 (0)		
Language proficiency [a]				
Spanish	6.26 (0.99)	6.25 (0.65)	0.02	0.98
English	3.95 (1.78)	3.91 (1.82)	0.48	0.64
Acculturation [b]				
American culture	2.60 (0.68)	2.57 (0.73)	0.13	0.89
Culture of origin	3.30 (0.46)	3.33 (0.45)	-0.38	0.71
Test score results				
WAT [c]	34.20 (9.58)	32.86 (8.22)	-0.05	0.95
Participant's memory functioning [d]	4.8 (1.7)	3.5 (2.52)	1.26	0.22
MMSE [e]	28.6 (1.4)	27.75 (1.89)	1.06	0.30
ST-DRS-2 [f]	133.2 (7.0)	126.75 (9.54)	1.59	0.12
Place of birth, no. (%)			0.82	0.98
Puerto Rico	9 (45)	2 (50)		
Colombia	5 (25)	1 (25)		
Cuba	3 (15)	1 (25)		
Argentina	1 (5)	0 (0)		
Peru	1 (5)	0 (0)		
Nicaragua	1 (5)	0 (0)		
US. Mainland	0 (0)	0 (0)		
Gross household annual income,			1.21	0.94
no. (%)	6 (30)	1 (25)		
<$25,000	6 (30)	1 (25)		
$25,000-49,999	3 (15)	1(25)		
$50,000-74,999	2 (10)	1 (25)		
$75,000-99,999	1 (5)	0 (0)		
$100,000-149,000	1 (5)	0 (0)		

>$150,000

Note. MMSE = Mini-Mental State Examination [16]; MSS = Memory Support System; ST-DRS-2 = Spanish Translation of the Dementia Rating Scale-Second edition [17]; WAT = Word Accentuation Test [29]. [a] Range is 1 to 7; higher score indicates greater self-report language proficiency. [b] Range is 1 to 4; higher score indicates greater self-report acculturation. [c] Range is 1 to 50; higher score indicates greater number of correctly read infrequent, prosodically accented Spanish words. [d] Range is 0 to 10; higher score indicates worse self-report memory functioning. [e] Range is 0 to 30; higher score indicates better global cognition. [f] Range is 0 to 144; higher score indicates better global cognition.
